# Unusual Presentation of Priapism Associated with Acute and Chronic Myeloid Leukemia in Two Patients: Emergency Management

**DOI:** 10.1155/2020/4982432

**Published:** 2020-08-12

**Authors:** Oumar Gaye, Ngor Mack Thiam, Ayun Cassell, Serigne Mourtalla Gueye, Yaya Sow, Boubacar Fall, Alain Khassim Ndoye

**Affiliations:** ^1^Urology-Andrology Department, Hôpital Aristide Le Dantec, Senegal; ^2^Urology-Andrology Department, Hôpital Général Idrissa Pouye, Senegal; ^3^Centre National de Transfusion Sanguine, Dakar, Senegal

## Abstract

Priapism is a rare urological emergency. It is rarely a telltale sign of myeloid leukemia. We report two cases of acute myeloid leukemia in a child and chronic myeloid leukemia in a young adult presenting with priapism. Puncture irrigation of the corpora cavernosa followed by systemic treatment to lower the hyperviscosity of the blood due to leukemia provided optimal outcome. Prompt emergency management is required to lower the complication of erectile dysfunction.

## 1. Introduction

Priapism is defined as the persistence of a complete or partial erection beyond four hours, after orgasm/cessation or without any sexual stimulation.

There are three different types of priapism: high-flow priapism, ischemic priapism, and recurrent priapism [1]. Ischemic priapism is the most frequent and serious type requiring emergency management to avoid complications such as erectile dysfunction. [1]. In the United States, ischemic priapism presents more commonly with an incidence of up to 5.34 per 100,000 men per year [2].

The hematological disorder is responsible to 20% of priapism [3]. Priapism resulting from acute or chronic myeloid leukemia is unusual [4]. In adult patients with leukemia, the incidence of priapism is estimated to be approximately 5% [5] and 15% in the pediatric population [6].

Emergency management requires combined systemic treatment along with local treatment of the corpora cavernosa [7]. We report two clinical cases of priapism occurring with of myeloid leukemia received in the urological emergency department.

## 2. Case 1

A case of a 46-year-old male patient was consulted for a painful erection for 48 hours without any previous sexual stimulation, trauma, or medication intake.

Physical assessment revealed a male with good general condition, grade 1 splenomegaly, hepatomegaly with hepatic notch at 14 cm, rigidity of the corpora cavernosa, and a flaccid glans penis.

The emergency laboratory workup showed Hemoglobin: 9.7 g/dl, Reds cells: 2920000/mm^3^, White Blood Cell: 526000/mm^3^, Platelets: 412000/mm^3^, Lactate Dehydrogenase: 1491 U/I, Urea: 0.23 g/L, and Creatinine level: 7.8 mg/L.

**Figure 1:**
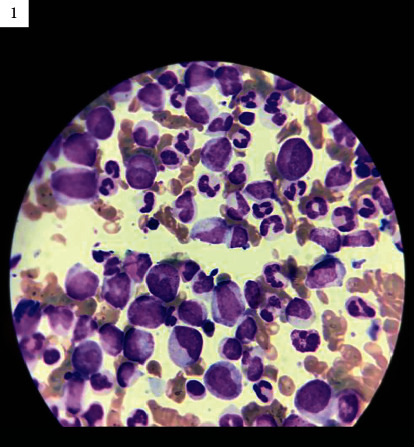
Blood smear showing myeloblast, promyelocytes, myelocytes, and eosinophil.

Blood smear (Figure 1) showed Myeloblast: 12%, Promyelocytes: 85%, Myelocytes: 11%, and Eosinophil: 4%.

The emergency management consisted of rehydration, an infusion of sodium bicarbonate, a puncture of the corpora cavernosa, injection of phenylephrine along with administration of hydroxycarbamide. Detumescence was achieved after 36 hours.

Subsequently, a myelogram showed bone marrow hyperplasia and a karyotype revealed a translocation between chromosomes 9 and 22 confirming the diagnosis of chronic myeloid leukemia. The patient was continued with doses of imatinib.

## 3. Case 2

A case of a 9-year-old child was seen in the emergency department for a sudden onset of a painful erection evolving for about 36 hours without any previous sexual stimulation, trauma, or medication intake.

**Figure 2:**
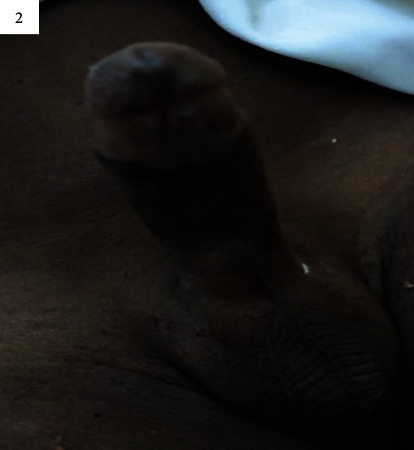
Rigidity of the corpora cavernosa and a flaccid glans penis.

The examination showed Pallor of the conjunctival mucosa, grade 1 splenomegaly, gingival enlargement, rigidity of the corpora cavernosa, and a flaccid glans penis (Figure 2).

The emergency laboratory workup: Hemoglobin: 3.4 g/dl, Reds cells: 1090000/mm^3^, White Blood Cell: 82000/mm^3^, Platelets: 81000/mm^3^, Neutrophils: 1% or 824/mm^3^, Eosinophils: 4% or 3296/mm^3^, Lymphocytes-h: 3% or 2472/mm^3^, Hematocrit: 11%, Urea: 0.21 g/L Creatinine level: 06 mg/L, and Blood smear: Blasts: 92%.

The emergency management consisted of penile skin refrigeration, rehydration, puncture of the corpora cavernosa with injection of phenylephrine, and induction of chemotherapy with vincristine and prednisolone, which achieved detumescence in 12 hours.

**Figure 3:**
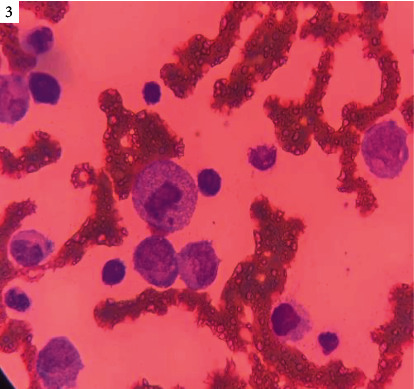
Bone marrow biopsy showing blast cell infiltration, leukemic infiltrate, mitoses, and presence of inflammatory cells.

The bone marrow biopsy performed later confirmed an acute myeloid leukemia (Figure 3). Hemoglobin electrophoresis was performed and ruled out sickle cell disease.

## 4. Discussion

Priapism is a rare urological emergency. Ischemic priapism results from an obstruction of the venous blood and is manifested by a painful erection with rigidity of the corpora cavernosa and a flaccid glans penis [7]. It is the most urgent and frequent type requiring emergency care to prevent complications such as erectile dysfunction.

To date, less than 20 cases of priapism and chronic myeloid leukemia [2, 3, 5, 8–15] have been reported and 2 cases of priapism and acute lymphoblastic leukemia [16, 17] (Table 1).

In Senegal, sickle cell disease is responsible for the majority of priapism cases [18]. Leukemia is rarely complicated by priapism. When it occurs, it is most often caused by chronic myeloid leukemia and more rarely by acute lymphoid leukemia or acute myeloid leukemia [9].

Hyperleukocytosis is considered the underlying pathology of priapism in these instances. The main mechanism is the aggregation of leukemia cells in the corpora cavernosa and the dorsal vein of the penis. Other accessory mechanisms are venous congestion of the corpora cavernosa secondary to mechanical pressure from the abdominal veins draining the spleen or infiltration of the sacral nerves or the central nervous system by leukemia cells [9].

Vadakan and Ortega described a case of priapism, which occurred from an acute lymphoid leukemia without hyperleukocytosis suggesting a disturbance of the central or peripheral nervous system [19]. Chang et al. have described priapism secondary to acute myeloid leukemia due to the direct penetration of the penis by leukemia cells [8]. As in most of the reports described [9–17], hyperleukocytosis was the most likely cause of priapism in the two cases of this report.

The American Urology Association [7] recommended combining systemic treatment of the organic cause with local treatment of the corpora cavernosa in cases of priapism secondary to leukemia. Systemic treatment is indicated for cytoreduction. It combines hydroxyurea with imatinib with or without leukapheresis to reduce hyperviscosity in chronic myeloid leukemia [8, 9, 20, 21] and chemotherapy in acute myeloid leukemia [22].

The local treatment at the level of the cavernous bodies involves a puncture evacuating the blood of the cavernous bodies along the lateral route at the penoscrotal or trans-glandular junction with an injection of alpha sympathomimetic agent. Amongst these agents, the use of etilefrine or phenylephrine has been favored because of their pure alpha-stimulating nature and better cardiac tolerance [21].

Other treatments such as radiotherapy of the penis, spinal anesthesia [23], irradiation of the spine [23, 24], the application of hot compresses [25], or penile refrigeration [19, 23] have been described with less satisfactory results.

The review showed that corporal aspiration/irrigation or a distal shunt was sufficient in achieving at least partial detumescence [2, 5, 8]. These procedures were followed by the treatment of the underlying leukemia with adequate hydration, hydroxyurea, imatinib, and leukapheresis [10–17]. The reports showed disease remission at a maximum 6-month follow-up with adequate erectile function.

## 5. Conclusion

Priapism is a usual presentation of myeloid leukemia and exceptionally rare association. A blood cell count showing hyperleukocytosis should suggest leukemia.

Emergent combination of systemic treatment with local treatment in the corpora cavernosa provides satisfactory results.

## Figures and Tables

**Table 1:** Literature review of priapism and leukemia from 2003 to 2020 using the PubMed database.

Series	Age	Diagnosis	Presenting symptoms	WBC cell/mm^3^	Platelet count cell/mm^3^	Treatment of priapism and leukemia	Outcome
Becerra-Pedraza et al. [2]	52 yrs	Chronic myeloid leukemia	Priapism for 144 hrs, fatigue, pallor	282 000		Corpora cavernosa drainage-irrigation penile shunts. Dasatinib	No ED on follow-up
Hazra et al. [3]	14 yrs	Chronic myeloid leukemia	Priapism for 24 hrs	226 900	310 000	Cavernosal aspiration and phenylephrine irrigation	No recurrence at 2-month follow-up
Khan et al. [5]	16 yrs	Chronic myeloid leukemia	Priapism for 264 hrs	614 800	7 090 000	Glans-cavernosal shunt hydroxyurea and allopurinol	Achieved detumescence. No info on ED
Nerli et al. [8]	19 yrs	Chronic myeloid leukemia	Priapism for 24 hrs	296 800	936 000	Corporal aspiration and phenylephrine irrigation hydroxyurea and imatinib	Achieved detumescence. No info on ED
Minckler et al. [9]	18 yrs	Chronic myeloid leukemia	Recurrent priapism	5 880 000	1 090 000	Hydroxyurea and imatinib	No info on follow-up
Chang et al. [10]	21 yrs	Chronic myeloid leukemia	Priapism for 19 hrs	216 800	1 746 000	Cavernosa aspiration and epinephrine irrigation hydroxyurea and interferon	No ED on follow-up
Qu et al. [11]	18 yrs	Chronic myeloid leukemia	Priapism for 72 hrs	257 000	5 450 000	Cavernosa-corpus spongiosum shunt, imatinib	No ED at 3-month follow-up
Yoshida et al. [12]	29 yrs	Chronic myeloid leukemia	Priapism for 48 hrs	263 000		Glans-cavernosal shunt hydroxyurea and imatinib	No ED at 5-month follow-up
Tazi [13]	33 yrs	Chronic myeloid leukemia	Priapism for 22 hrs	400 000	1 200 000	Cavernosa aspiration and epinephrine irrigation	No recurrence on follow-up
Ponniah et al. [14]	19 yrs	Chronic myeloid leukemia	Priapism for 18 hrs	513 000		Failed cavernosal aspiration + leukapheresis	No ED on follow-up
Ergenc et al. [15]	18 yrs	Chronic myeloid leukemia	Priapism for 72 hrs	100 000	1 002 000	İmatinib, allopurinol + leukapheresis	No ED at 6-month follow-up
Gupta et al. [16]	10 yrs	Acute lymphoblastic leukemia	Priapism, fever, headache	693 000	40 000	Corporal aspiration followed by phenylephrine irrigation, allopurinol + steroids	Died after 48 hours
Güzelküçük et al. [17]	9 yrs	Acute lymphoblastic leukemia	Priapism for 18 hrs	583 000	51 000	Corporal aspiration followed by hydroxyurea, allopurinol + leukapheresis	Achieved detumescence

ED: erectile dysfunction; Hrs: hours; WBC: White Blood Cell; Yrs: years.
